# CD16 and Siglec expression refine the phenotypic heterogeneity of steady-state myeloid-derived suppressor cells

**DOI:** 10.3389/fonc.2025.1570121

**Published:** 2025-06-23

**Authors:** Chris D. St. Laurent, Zeinab Jame-Chenarboo, Alyssa E. Beck, Stacey Stubblefield, Shiteng Duan, Darren Sigal, Matthew S. Macauley

**Affiliations:** ^1^ Department of Chemistry, University of Alberta, Edmonton, AB, Canada; ^2^ Scripps Clinic and Scripps Cancer Center, San Diego, CA, United States; ^3^ Department of Molecular Medicine, Scripps Research Institute, San Diego, CA, United States

**Keywords:** Siglec, MDSC, neutrophil, CD16, T cell suppression, steady-state

## Abstract

**Background:**

Polymorphonuclear myeloid-derived suppressor cells (PMN-MDSCs) mediate cancer immune suppression by promoting an immunosuppressive microenvironment that inhibits effective anti-tumor immunity. However, they are still a poorly understood, heterogeneous mix of neutrophil subsets. This study aims to determine the Siglec expression profile on several neutrophil subsets and assess their immunosuppressive ability.

**Methods:**

We identified CD16^high^ and CD16^low^ neutrophil subsets from the low-density fractions of human peripheral blood and compared them to high-density neutrophils. We profiled the expression of the entire family of Siglecs on these three key neutrophil populations under steady-state conditions in healthy subjects as well as cancer patients. Moreover, the ability of these populations, isolated from healthy subjects, to suppress T cell proliferation was assessed.

**Results:**

Two distinct subpopulations were investigated within the low-density fraction of human peripheral blood (CD15^+^CD66b^+^CD16^low^ and CD15^+^CD66b^+^CD16^high^) and compared to high-density neutrophils (CD15^+^CD66b^+^CD16^high^). We found that in addition to CD33 (Siglec-3), Siglec-5/-14, -7, and -9, are differentially expressed on the CD16^low^ and CD16^high^ low-density subsets in both healthy, steady-state subjects, and cancer patients. Upregulated expression of CD33 on the CD16^low^ cells led to the initial speculation that they are MDSCs. As the differential expression of Siglec-9 between these two populations was striking, we used CD16 and Siglec-9 double staining to quantify these populations, which demonstrated that the CD16^low^Siglec-9^low^ population is greatly upregulated in cancer patients. The CD16^high^ low-density and high-density neutrophils, but not the CD16^low^ low-density neutrophils from healthy subjects, inhibited T cell proliferation, indicating that the CD16^low^Siglec-9^low^ population are not MDSCs.

**Conclusions:**

These results demonstrate that Siglecs are differentially expressed on neutrophil subsets, and along with CD16, may be used to help further define what is a PMN-MDSC. Consistent with current observations by others, PMN-MDSCs may encompass an array of neutrophil subtypes, including low-density neutrophils, and point to the need for more work to precisely define the genetic signatures of PMN-MDSCs.

## 1 Introduction

The tumor microenvironment is composed of cellular and soluble immunosuppressive components that promote tumor growth and immune evasion (1, 2). The cellular components of the suppressive tumor microenvironment contain different types of cells, such as tumor associated macrophages (TAM) and tumor associated neutrophils (TAN) (3), which are thought to be derived from peripheral blood monocytes (4) and neutrophils (5), respectively. Another putative cell type implicated in immune suppression are myeloid-derived suppressor cells (MDSCs) (6, 7). MDSCs are proposed to be immunosuppressive through multiple mechanisms (8–11). Despite the extensive number of publications characterizing MDSCs, the precise population that represents these cells is still not resolved (12). It is widely believed that MDSCs are upregulated in cancer (13), and other pathological conditions (14) and the vast majority of studies have isolated MDSCs from cancer patients but not healthy subjects. This may be due to low numbers of MDSCs in healthy, steady-state conditions, making it challenging to study this population. However, more recently, there has been a call for further study of MDSCs in steady-state, or homeostatic conditions (12).

MDSCs have historically been divided into two main subsets: polymorphonuclear MDSCs (PMN-MDSC) and monocytic MDSCs (M-MDSC). PMN-MDSCs share many of the same markers as neutrophils (CD11b^+^CD33^mid^CD14^-^CD15^+^CD66b^+^HLA-DR^-^), while M-MDSCs are phenotypically described more like monocytes (CD11b^+^CD33^high^CD14^+^CD15^-^HLA-DR^-/low^). In recent years, researchers have begun to refine what constitutes an MDSC (15, 16), although it is important to note that there is still no consensus (12). For PMN-MDSCs, the majority of studies use density centrifugation to separate the less dense peripheral blood mononuclear cell (PBMC) fraction from the heavier granulocyte fraction, with the PMN-MDSCs generally considered to be part of the low-density neutrophils. However, even within this low-density fraction, there are conflicting reports on what subpopulations of neutrophils are immunosuppressive and whether PMN-MDSCs may simply be immature neutrophils (17, 18) or degranulated neutrophils (7, 16, 19). For PMN-MDSCs to be a defined population, experts agree that a more robust panel of markers is needed (12).

Three markers that have been explored for their ability to subset the low-density neutrophils, in hopes of better defining PMN-MDSCs, are CD16, LOX-1, and CD33 (Siglec-3). During granulopoiesis, maturing neutrophils start to express CD16 (7), and several studies have used this marker to help differentiate PMN-MDSCs from mature and immature neutrophils (20, 21). LOX-1 is reported to be expressed at low levels on mature neutrophils, and high on PMN-MDSCs, therefore, LOX-1 has been proposed to differentiate these two populations (18). CD33 is another marker commonly used to help identify MDSCs but is mainly used as a general marker of myeloid cells and used early in the gating process to differentiate between M-MDSCs (CD33^high^) and PMN-MDSCs (CD33^mid^) (22–24). While CD33 is reported to be expressed at higher levels on PMN-MDSCs compared to mature neutrophils (7, 15, 25), CD33 is also highly expressed on immature neutrophils (26), so it remains to be determined if CD33 levels differentiate PMN-MDSCs from immature neutrophils.

CD33 is a member of the Siglecs (Sialic acid-binding immunoglobulin type lectins), which are a family of immunomodulatory receptors that regulate immune cell signaling through interactions with their sialic acid-containing glycoconjugate ligands (27). Sialic acid is considered as a self-associated molecular pattern as it is not presented by most bacteria and fungi (28). There are 15 Siglecs in humans with diverse and broad expression on different immune cells. A majority of Siglecs have an immunoreceptor tyrosine-based inhibitory motif (ITIM) within an intercellular tail that can antagonize immune cell signaling. Siglecs expressed on immune cells serve as immune check points and dampen the immune response through interactions with their Sialic acid ligands (29). Recently, a functional role for another member of the Siglec family, Siglec-9, has been described on PMN-MDSCs (30). Another study analyzed Siglec expression on PMN-MDSCs and M-MDSCs, but how the cells in that study differed from conventional neutrophils and monocytes is not clear (31). As the differential expression of Siglecs are broadly used to define immune cell subsets (27), it is possible that differential expression of Siglecs could help differentiate neutrophil subsets including mature high-density neutrophils, low-density neutrophils, and immature neutrophils, of which at least one of these are expected to have immunosuppressive properties characteristic of PMN-MDSCs.

Here, we assessed the expression of Siglecs on each neutrophil subset as well as assessed their ability to suppress T cell proliferation. We find that CD15^+^CD66^+^ neutrophils from the PBMCs of both healthy, steady-state subjects, and cancer patients, differentially express CD33, Siglec-5/-14, -7, and -9 based on the expression of CD16. Higher expression of CD33, along with lower expression of Siglec-9, on the CD16^low^ low-density neutrophils led us to speculate that these are PMN-MDSCs. Supporting this hypothesis is our observation that the number of CD15^+^CD66b^+^CD16^low^Siglec-9^low^ cells within the PBMCs increased twelve-fold in cancer patients compared to healthy controls. However, we found that the CD16^high^ low-density neutrophils, and not the CD16^low^ population, inhibited T cell proliferation in healthy subjects. Moreover, the CD16^high^ low-density neutrophils suppressed T cell proliferation to the same degree as CD16^high^ high-density neutrophils. Overall, these results demonstrate that Siglecs are differentially expressed on neutrophil subsets and point to none of the low-density neutrophil subsets having a more profound immunosuppressive capacity than mature neutrophils.

## 2 Methods

### 2.1 Human subjects

Peripheral blood from gastrointestinal (GI) cancer patients was obtained at Scripps Health, San Diego, CA, under IRB protocol number IRB-15-6598, and analyzed at the University of Alberta with approval from the Human Ethics Research Board – Biomedical Panel; study number Pro00083934. Blood was collected from pancreatic, colorectal, esophageal, and cholangiocarcinoma patients with a range of severity from Stage II to stage IV.

Blood from healthy donors was collected at the University of Alberta, Edmonton, AB, Canada, with approval from the Human Research Ethics Board – Biomedical Panel, study number Pro00092144. All study participants gave written informed consent.

### 2.2 Blood collection and processing

Peripheral blood was collected from GI cancer patients via venipuncture and collected into BD Vacutainer tubes containing EDTA. Blood was stored at 4°C and shipped overnight to the University of Alberta in Therapak® NanoCool temperature-controlled shipping boxes (Avantor) to maintain 2°C-8°C. Blood from healthy donors was collected into similar EDTA vacutainers and processed either immediately or stored overnight at 4°C to match cancer blood sample storage conditions. Both healthy and cancer blood was then analyzed together.

5 mL whole blood was diluted into 15 mL HBSS, layered onto 15 mL of Ficoll-Paque^TM^ Plus (Cytiva) and spun at 400 RCF for 40 minutes, at 18°C. The interface, containing the PBMCs, and the granulocyte layer were collected separately, washed with HBSS, and the red blood cells (RBC) were lysed. Briefly, cells were resuspended in 40 ml RBC lysis buffer (80.2 g/L ammonium chloride, 8.2 g/L sodium bicarbonate, 3.7 g/L EDTA) for 2 minutes, spun at 400 RCF for 5 minutes, then washed with HBSS. Cells were then resuspended in 0.5 mL flow buffer (HBSS containing 0.1% BSA and 0.1% EDTA).

### 2.3 Antibody staining and flow cytometry

Cells were first incubated with Human TruStain FcX Fc receptor blocking solution (BioLegend) at 1:200 for 10 minutes at rt. Cells were then stained with a mixture of antibodies for 30 minutes, on ice. Antibodies used were: CD3-FITC (BD, clone UCHT1, 1:100), CD19-APC/Cy7 (BD, 1:100), CD56-BV510 (BD, clone NCAM16.2, 1:100), CD66b-BV421 (BD, clone G10F5, 1:100), CD123-PE/Cy7 (BioLegend, clone 6H6, 1:100), CD16-BV786 (BD, clone 3G8, 1:100), Siglec 9-PE (BioLegend, clone K8, 1:200), CD14-BV605 (BD, clone M5E2, 1:100), CD15-BUV395 (BD, clone HI96, 1:100), and LOX-1-APC (Biolegend, clone 15C4, 1:100). Cells were then washed with flow buffer twice and resuspended in 0.3 mL flow buffer containing the live/dead marker 7-aminoactinomycin D (7-AAD) (Thermo Fisher Scientific). Flow cytometry was done using a BD LSRFortessa X-20 flow cytometer. For each sample, the entire 0.3 mL was run, and both cell numbers in target gates and CountBright™ bead numbers were recorded. To calculate total cell numbers, 10,000 CountBright™ beads (Thermo Fisher Scientific) were added to each sample prior to flow cytometry and the number of cells was calculated based on the number of beads recovered and expressed as cells/mL of blood.

For Siglec-staining, each blood sample was divided into 13 aliquots and stained as above, however each Siglec antibody (Siglec-1, -2, -3, -4, -5/-14, -6, -7, -8, -9, -10, and isotype) was added (BioLegend, 1:200) in the PE channel instead of Siglec-9. For Siglec-11 and -15, a goat anti mouse total IgG secondary antibody conjugated to PE was used (1:500).

### 2.4 T cell suppression assay

PBMCs and granulocytes were isolated from healthy subjects, as above, and the PBMC CD15^+^ population was enriched using CD15 Microbeads (Miltenyi Biotec) as per the manufacturer’s protocol. Enriched PBMCs, and granulocytes, were then stained for 30 minutes on ice with the following antibodies (detailed above, unless otherwise indicated): CD56-BV510, CD3-FITC, CD19-APC/Cy7, CD123-PE/Cy7, CD16-BV786, CD14-BV421 (BioLegend, clone M5E2), CD15-APC (BioLegend, clone HI98), all at 1:100, and Siglec 9-PE (1:200). The CD15^+^CD16^low^Siglec-9^low^ and CD15^+^CD16^high^Siglec-9^high^ populations from enriched PBMCs and the CD15^+^CD16^high^ population from granulocytes was sorted using a BD FacsMelody cell sorter.

Autologous human T cells were isolated based on the manufacturer’s protocol using the human Pan T cell isolation kit (Miltenyi Biotec). Isolated T cells were resuspended in pre-warmed PBS and stained with 5 μM cell trace violet (CTV, Thermo Fisher Scientific) for 6 min at 37°C in the dark. 5 mL of pre-warmed RPMI media containing 10% FBS was added to the T cells and then incubated for two minutes at rt to remove unbound CTV. 1 mL of pre-warmed RPMI (10% FBS, 100 Units/mL Penicillin, 100 μg/mL Streptomycin, 0.02% βME, 1% HEPES, 1% Sodium pyruvate, 1% MEM non-essential amino acids) was then added to the T cells. Cells from each donor were counted and human anti-CD3/CD28 dynabeads (Thermo Fisher Scientific) were added based on the manufacturer’s protocol (1:1 bead:T cell). T cells were cultured in a 96-well round bottom plate at a density of 5,000 cells/well in the above-mentioned media with 100 U/mL IL-2 (BioLegend).

Sorted low-density PBMCs and high-density neutrophils were added to the T cells at 4:1, 2:1, 1:1, and 1:2 ratios and incubated together for 96 hours. T cell proliferation was measured by tracking CTV dilution using a BD LSRFortessa X-20 flow cytometer using the following antibodies at 1:150: CD4-BV711 (BioLegend, Clone RPA-T4), CD8a-FITC (BioLegend, Clone HIT8a), CD15-APC (BioLegend, Clone HI98), CD3-BV650 (BioLegend, Clone OKT3) and 7-AAD as a viability dye. Proliferation data was plotted using the measured Proliferation Index from FlowJo software (Becton Dickinson, BD).

### 2.5 Statistical analysis

**Figure 1:**
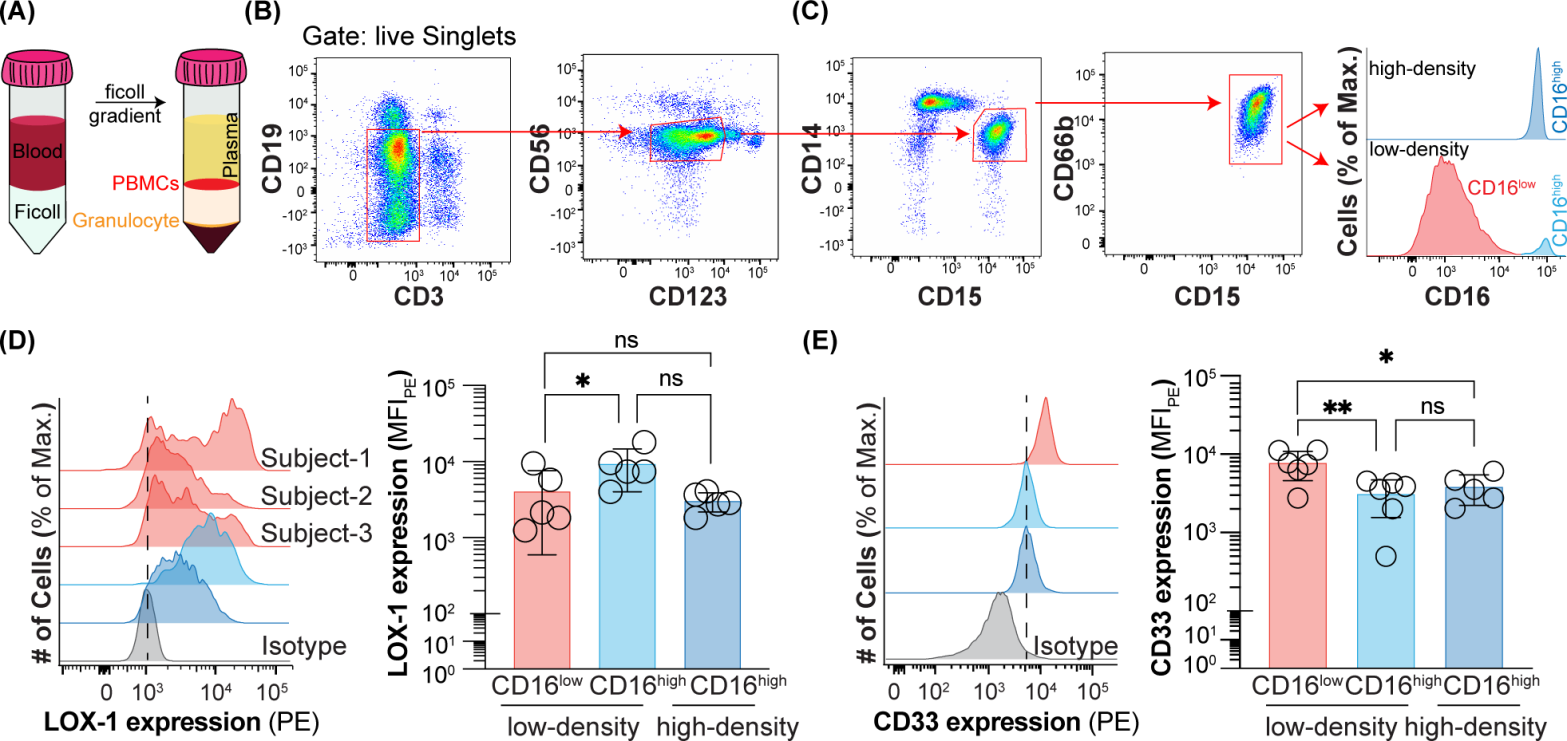
Expression of CD16, CD33, and LOX-1 on peripheral blood neutrophils from healthy subjects. **(A)** Schematic of ficoll gradient for separating low-density and high-density neutrophils. **(B)** Flow cytometry gating strategy for gating out B cells, T cells, NK cells, basophils and monocytes. **(C)** Flow cytometry gating strategy for CD16^low^ low-density, CD16^high^ low-density, and CD16^high^ high-density neutrophils. **(D)** LOX-1 expression on CD16^low^ low-density, CD16^high^ low-density, and CD16^high^ high-density neutrophils. Data is presented as median fluorescent intensity of LOX-1 expression and representative histograms for CD16^high^ low-density and CD16^high^ high-density neutrophils and histograms of CD16^low^ low-density for three healthy subjects. **(E)** CD33 expression on CD16^low^ low-density, CD16^high^ low-density, and CD16^high^ high-density neutrophils. Data is presented as representative histograms and median fluorescent intensity of CD33 expression. * = p≤0.05, ** = p≤0.01, ns = not significant.

**Figure 2:**
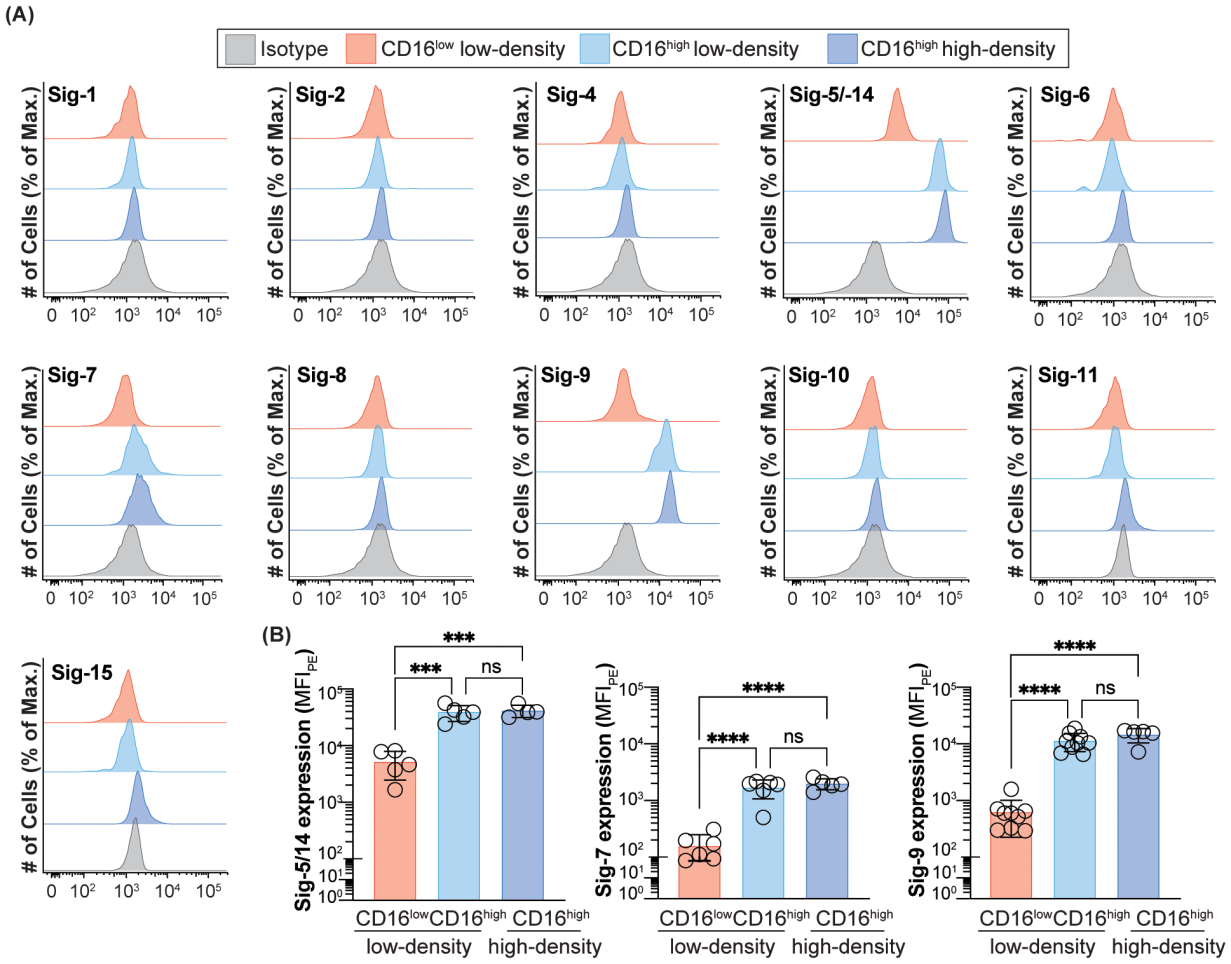
Expression of Siglecs on peripheral blood neutrophils in healthy subjects. **(A)** Expression of Siglec-1, -2, -4, -5/-14, -6, -7, -8, -9, -10, -11 and -15 on CD16^low^ low-density, CD16^high^ low-density, and CD16^high^ high-density neutrophils. Data is presented as representative flow cytometry histograms. **(B)** Quantification of Siglec-5/-14, -7 and -9 expression CD16^low^ low-density, CD16^high^ low-density, and CD16^high^ high-density neutrophils. Data is presented as median fluorescent intensity of Siglec expression. *** = p≤0.001, **** = p≤0.0001, ns = not significant.

**Figure 3:**
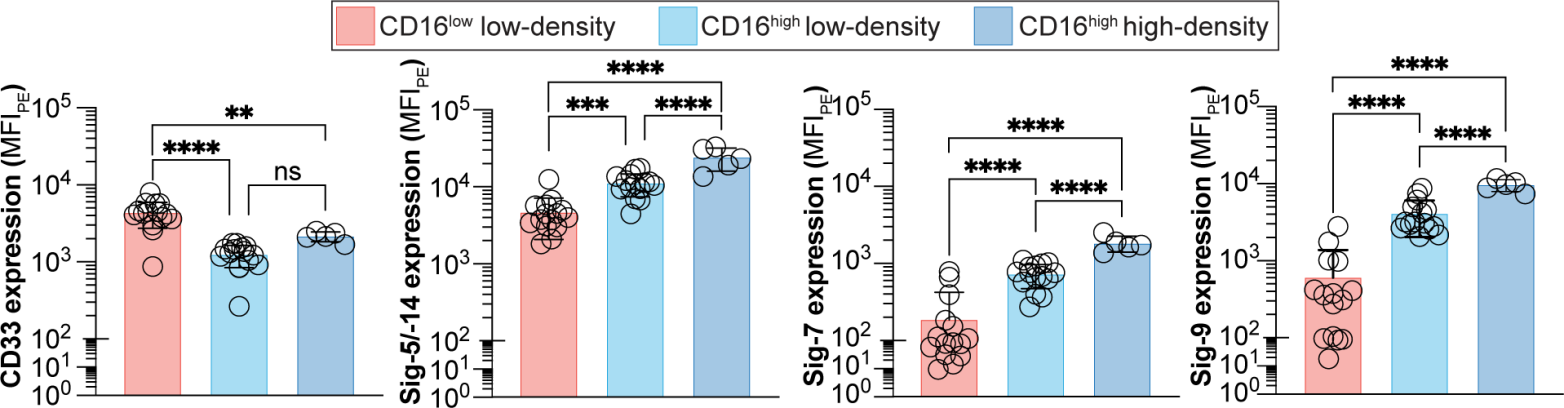
Expression of Siglecs on peripheral blood neutrophils from cancer patients. Expression of Sigec-3, -5/-14, -7 and -9 on CD16^low^ low-density, CD16^high^ low-density, and CD16^high^ high-density neutrophils. Data is presented as median fluorescent intensities of Siglec expression. ** = p≤0.01, *** = p≤0.001, **** = p≤0.0001, ns = not significant.

**Figure 4:**
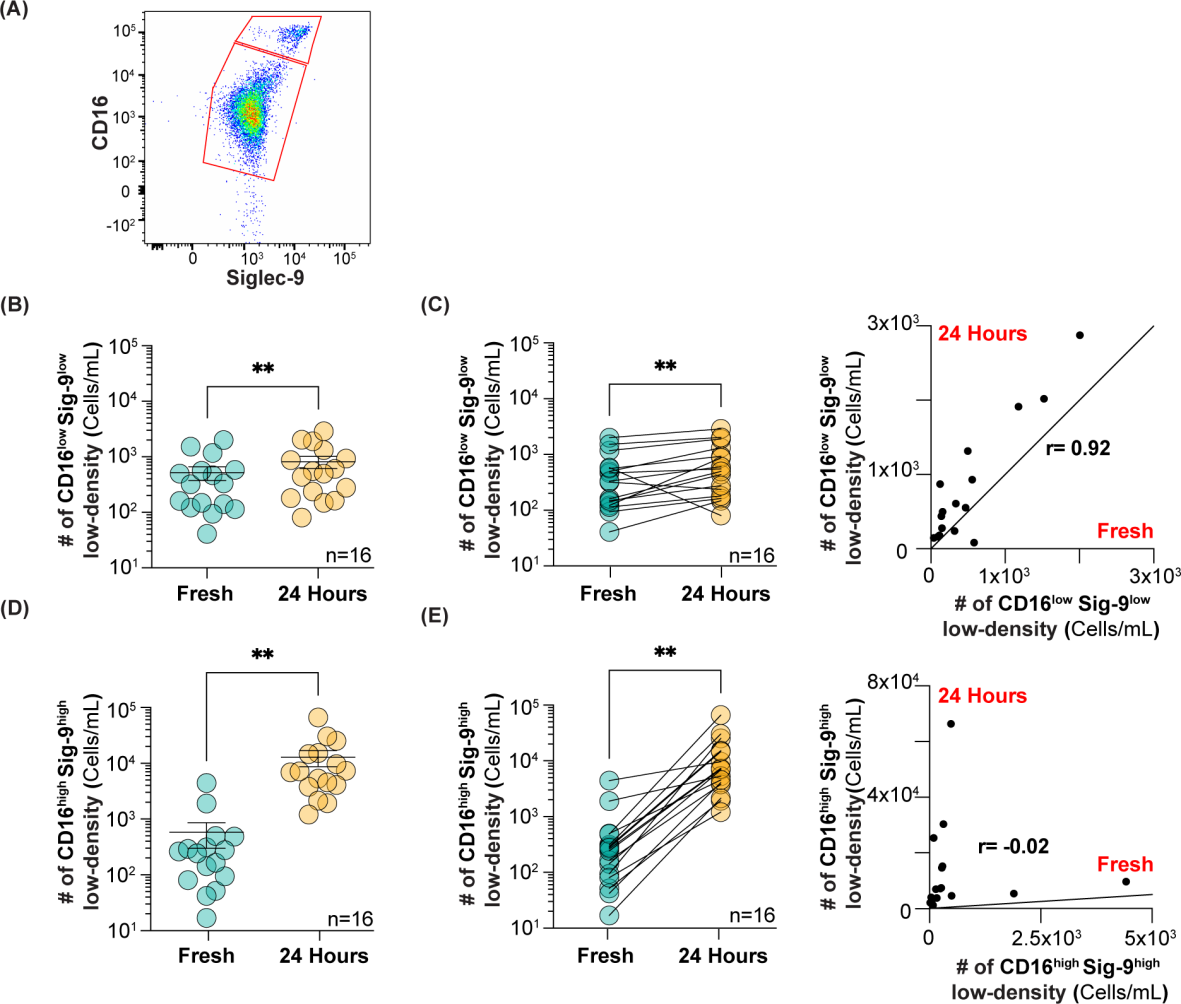
Analysis of CD16^low^Siglec-9^low^ and CD16^high^Siglec-9^high^ low-density neutrophils after 24 h at 4°C. **(A)** Flow cytometry gating strategy for using Siglec-9 to separate CD16^low^Siglec-9^low^ and CD16^high^Siglec-9^high^ neutrophils. **(B)** Number of CD16^low^Siglec-9^low^ low-density neutrophils isolated fresh and after 24 h. **(C)** Correlation between CD16^low^Siglec-9^low^ low-density neutrophils in fresh *vs* 24 h. **(D)** Number of CD16^high^Siglec-9^high^ low-density neutrophils isolated fresh and after 24 h. **(E)** Correlation between CD16^high^Siglec-9^high^ low-density neutrophils in fresh *vs* 24 h. ** = p≤0.01.

**Figure 5:**
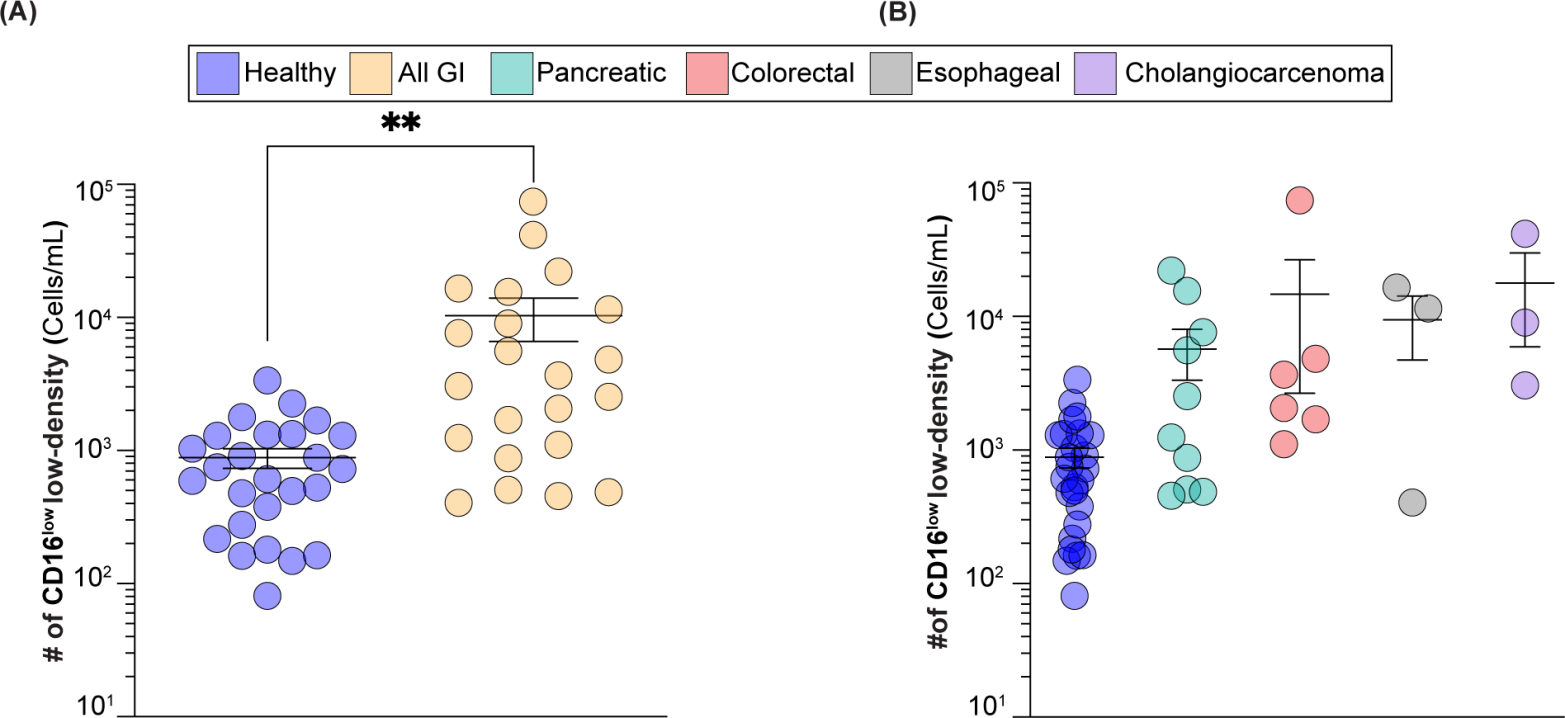
CD16_low_ low-density neutrophils numbers in GI cancer patients *vs* healthy subjects. **(A)** Number of CD16^low^ low-density neutrophils in healthy subjects *vs* GI cancer patients. **(B)** Number of CD16^low^ low-density neutrophils in healthy group *vs* GI cancer patients broken down into colorectal, esophageal, cholangiocarcinoma, and pancreatic cancers. ** = p≤0.01.

**Figure 6:**
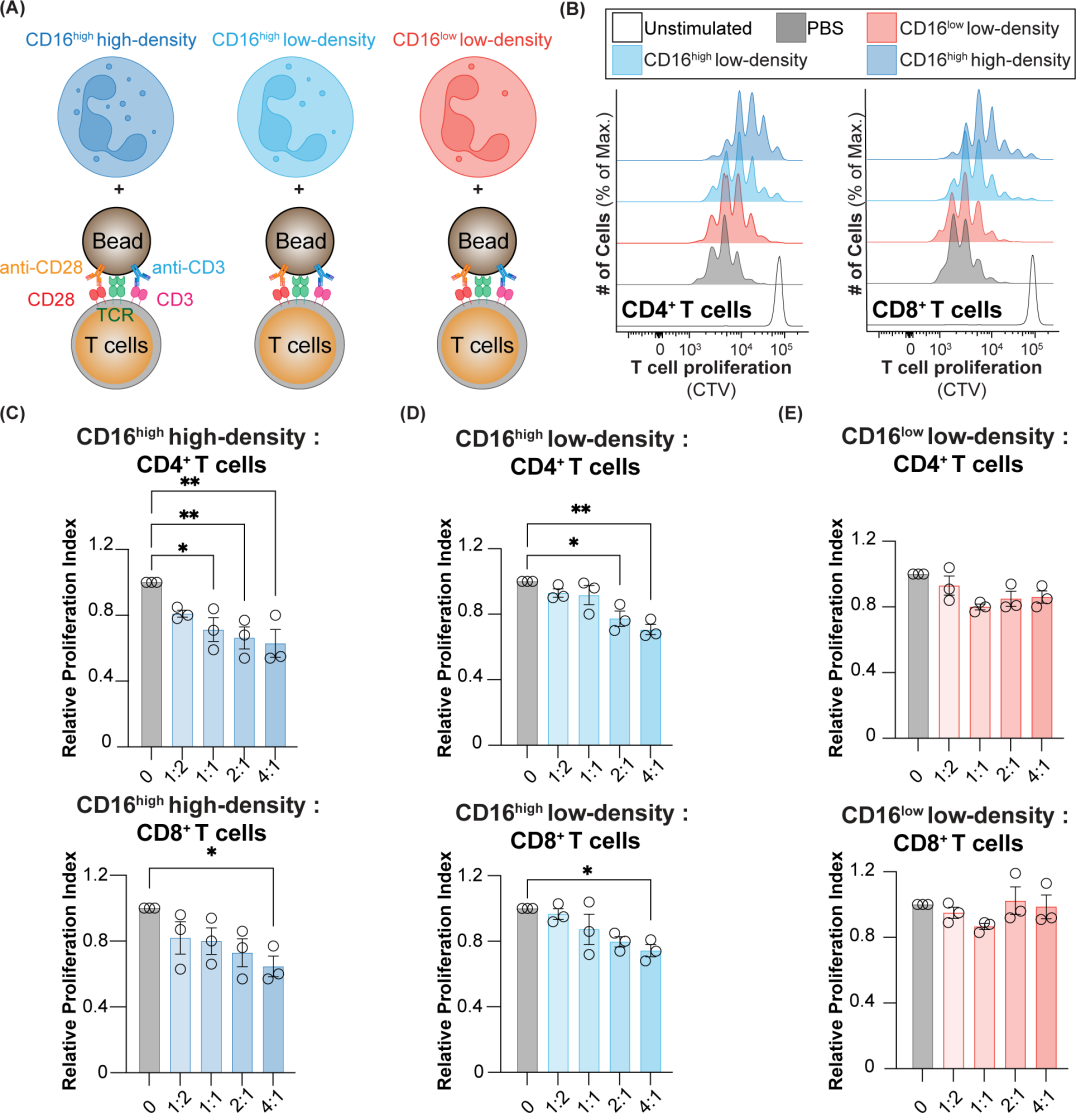
T cell suppression using CD16^low^ and CD16^high^ low-density neutrophils, and CD16^high^ high-density neutrophils. **(A)** Schematic of neutrophils co-cultured with T cells that were activated with anti-CD3/28 beads. **(B)** Representative histograms of CD4^+^ and CD8^+^ T cell proliferation when co-cultured with PBS, CD16^low^ and CD16^high^ low-density neutrophils, and CD16^high^ high-density neutrophils. Relative proliferation index of CD4^+^ and CD8^+^ T cells co-cultured with **(C)** CD16^high^ high-density neutrophils, **(D)** CD16^high^ low-density neutrophils, and **(E)** CD16^low^ low-density neutrophils. * = p≤0.05, ** = p≤0.01.

Statistical analysis was done using one-way ANOVA and Tukey multiple comparison post-test in **Figures 1**–**3**, **5**, **6** using GraphPad Prism 9 software (Dotmatics). A two-tailed paired *t*-test was used in **Figure 4**, and the Pearson correlation coefficient was calculated.

## 3 Results

### 3.1 Siglec expression on human peripheral blood CD15^+^CD66b^+^ low-density neutrophils in healthy subjects

Human peripheral blood low-density neutrophils were separated from high-density neutrophils using a Ficoll gradient, where they are present in the PBMC and granulocyte fractions, respectively (**Figure 1A**). A multicolor panel of antibodies was used to identify CD15^+^CD66b^+^ cells within the PBMCs by first gating out other cell types with standard lineage markers: B (CD19) and T cells (CD3), NK cells (CD56), basophils (CD123), and monocytes (CD14) (**Figure 1B**). The remaining cells were predominantly CD15^+^CD66b^+^ cells of the neutrophil lineage. Within the PBMCs, but not the granulocytes, these cells contained CD16^high^ and CD16^low^ subpopulations (**Figure 1C**). Differential expression of CD16 on low-density neutrophils has been observed previously (20, 21, 32). To carefully assess how the other two described markers of PMN-MDSCs, LOX-1 and CD33, track with CD16 expression, we assessed their levels in the CD16^high^ and CD16^low^ low-density neutrophils. The CD16^high^ population did express higher levels of LOX-1 than the CD16^low^ population of low-density neutrophils, despite substantial heterogeneity in the CD16^low^ population between individuals (**Figure 1D**). Consequently, there was no difference in the expression of LOX-1 between CD16^high^ low- and high-density neutrophils. On the other hand, there was a very consistent inverse correlation between CD16 and CD33 expression levels (**Figure 1E**). As higher levels of CD33 expression have been reported to be associated with PMN-MDSCs, compared to neutrophils, it was interesting to observe that CD33 expression levels were indistinguishable between high-density neutrophils and CD16^high^ low-density neutrophils, but markedly increased on the CD16^low^ low-density neutrophils. These results suggested that the CD16^low^ low-density neutrophils could be PMN-MDSCs.

Given that CD33 is differentially expressed on CD16^low^ *vs* CD16^high^ low-density neutrophils, we wondered if any other Siglecs are likewise differentially expressed. Accordingly, we assessed the expression of the entire Siglec family on the CD16^low^ *vs* CD16^high^ subpopulation of CD15^+^CD66b^+^ cells from the PBMCs in a cohort of healthy subjects, with additional comparison to Siglec expression on CD15^+^CD66b^+^ high-density neutrophils from the granulocytic fraction. Only CD33, Siglec-5/-14, -7, and -9 showed significant expression levels (**Figure 2A**). Similar to CD33, the other three Siglecs (Siglec-5/-14, -7, and -9) displayed significant differences in expression levels between the CD16^low^ and CD16^high^ low-density neutrophils, while there were no differences in expression between the CD16^high^ low-density and high-density neutrophils (**Figures 2A, B**). However, unlike CD33, which had higher expression on the CD16^low^ subpopulation (**Figure 1E**), Siglec-5/-14, -7, and -9, showed significantly reduced expression on the CD16^low^ cells in the PBMCs.

### 3.2 Siglec expression on low-density neutrophils in gastrointestinal cancer patients

To examine if the expression profile of Siglecs between the CD16^low^ and CD16^high^ low-density neutrophils are altered in cancer, we examined Siglec expression on cells obtained from peripheral blood of GI cancer patients (**Figure 3**). Consistent with the healthy group, CD33, Siglec-5/14, -7, and -9 were all significantly different between the CD16^low^ and CD16^high^ subpopulations. Specifically, CD33 expression was elevated on the CD16^low^ low-density neutrophils compared to the CD16^high^ low-density neutrophils, while the other three Siglecs showed the opposite pattern. Unlike in healthy subjects, there is a small, but statistically significant, difference in Siglec-5/-14, -7, and -9 expression between the CD16^high^ low-density neutrophils and high-density neutrophils.

### 3.3 Quantifying CD16^low^ low-density neutrophils in GI cancer patients

It is well documented that PMN-MDSCs are increased in many types of cancers (33), including pancreatic and other GI cancers (34, 35). Therefore, we were interested to see if the CD16^low^ low-density neutrophils were increased in cancer patients. As we did not have access to fresh blood from cancer patients, we carefully assessed whether storing whole blood overnight at 4°C for analysis the next day, to mimic an overnight shipment on ice, altered the number of CD16^low^ and CD16^high^ low-density neutrophils. To improve the separation of CD16^low^ and CD16^high^ populations for quantification, we included Siglec-9 in our staining protocol (**Figure 4A**), as this marker showed the greatest difference between CD16^low^ and CD16^high^ populations; 13-fold higher in healthy subjects (**Figure 2B**), and 7-fold higher in cancer patients (**Figure 3**). Although there was a statistically significant increase in the absolute number of CD16^low^Siglec-9^low^ low-density neutrophils after 24 h, the average increase was modest, from 520 to 820 cells/mL (**Figure 4B**). The Pearson correlation coefficient between fresh and 24 h blood was 0.92 (**Figure 4C**). In contrast, the number of CD16^high^Siglec-9^high^ low-density neutrophils greatly increased after 24 h, from 580 to 12,900 cells/mL (**Figure 4D**), with a Pearson correlation coefficient of -0.21 (**Figure 4E**). These results suggest that quantifying the number of CD16^low^Siglec-9^low^, but not CD16^high^Siglec-9^high^, low-density neutrophils from whole blood stored overnight at 4°C may provide an accurate gauge of their numbers in fresh blood.

Blood from healthy subjects (n=26) and GI cancer patients (n=22) was analyzed for the number of CD16^low^ low-density neutrophils. In healthy subjects, CD16^low^ numbers ranged from 80 to 3,400 cells/mL, with an average of 880 cells/mL. In cancer patients, the range of these cells was from 400 to 74,500 cells/mL, with an average of 10,300 cells/mL (**Figure 5A**), which represents a statistically significant twelve-fold increase. As the GI cancer group included pancreatic, colorectal, esophageal, and cholangiocarcinoma cancers, we broke down our findings into individual groups and while not statistically significant, likely due to fewer number of patients in each group, there was a clear trend towards elevated CD16^low^Siglec-9^low^ low-density neutrophils in these cancers (**Figure 5B**).

### 3.4 CD16^high^ but not CD16^low^ low-density neutrophils have suppressive activity in healthy subjects

The definition of a PMN-MDSC relies on their ability to suppress T cell activity. Accordingly, we assessed whether the three populations of neutrophils (CD16^low^ and CD16^high^ low-density neutrophils, and high-density neutrophils) can suppress T cell proliferation. As we have shown that the CD16^high^Siglec-9^high^ population from cancer blood assayed at 24 h cannot be reliably isolated, we assessed T cell suppression in healthy, steady-state subjects. The three neutrophil populations were isolated by fluorescence-activated cell sorting and mixed with autologous T cells at various ratios to assess their ability to suppress the proliferation of CD4^+^ and CD8^+^ T cells (**Figures 6A, B**). We found that high-density neutrophils had the strongest ability to inhibit T cell proliferation, with statistically significant suppression of CD4^+^ T cells down to a ratio of 1:1 neutrophils:T cells, and at a 4:1 ratio for CD8^+^ T cells (**Figure 6C**). CD16^high^ low-density neutrophils also significantly suppressed CD4^+^ and CD8^+^ T cells but for CD4^+^ T cells, suppression was only observed down to a 2:1 neutrophil:T cell ratio, and a 4:1 ratio for CD8^+^ T cells (**Figure 6D**). On the other hand, CD16^low^ low-density neutrophils did not show any suppression of either CD4^+^ and CD8^+^ T cells (**Figure 6E**). Taken together, these results show that CD16^low^ low-density neutrophils have no suppressive activity and, therefore, are likely not PMN-MDSCs. On the other hand, the ability of CD16^high^ low-density neutrophils to suppress T cells gives them the key phenotypic property of a PMN-MDSC. Nevertheless, high-density neutrophils and CD16^high^ low-density neutrophils had similar suppressive activities, bringing into question of what precisely constitutes a PMN-MDSC.

## 4 Discussion

Since the early 2000’s, researchers have been trying to characterize and define an MDSC, but a survey of the literature reveals that while they can be defined phenotypically via their suppressive activity, markers to precisely define them are lacking. Accordingly, there has been a concerted effort to unify the definition and markers needed to define a PMN-MDSC, with limited success (11, 16). More recently, a panel of experts gave their views on the challenges of defining MDSCs and their consensus is that MDSCs are a heterogeneous population of cells of the neutrophil lineage, of various maturation states, but that we still do not have a panel of markers that can unequivocally define what is an MDSC (12). Using our panel of markers, including CD16 and Siglec-9, which are not commonly used by other groups, we feel is a step in the right direction in helping to better define what a PMN-MDSC is, and is not.

The general consensus in the literature has been that PMN-MDSCs are not very abundant in healthy subjects, in a steady-state environment (13), however, few studies have isolated PMN-MDSCs from healthy subjects and assessed their ability to suppress T cell proliferation. It is, therefore, interesting that high-density neutrophils from healthy subjects were able to suppress T cell proliferation in our assays, along with the CD16^high^ low-density population. Several other studies have shown similar results of T cell inhibition by high-density neutrophil populations in both healthy subjects (20), and cancer patients (36) but, unfortunately, the majority of published studies do not include high-density neutrophil controls. Moreover, in studies that do not fractionate neutrophils based on density, the contribution of mature neutrophils to their PMN-MDSC suppressive population may not be accounted for, and even in studies that do fractionate based on density, contamination of the low-density fraction by mid- or high-density neutrophils could still be an issue, and additional neutrophil maturity markers on these populations could be assessed to rule this out. Although we found the CD16^high^ low-density population in healthy subjects to be suppressive, the degree of suppression is relatively low compared to some studies on cancer patients (37, 38), albeit some studies have observed similar levels of suppression as in our study (39). This could indicate that while PMN-MDSCs can indeed be found in steady-state, healthy subjects, they are not yet primed to suppress T cells to as great a degree as in the pathogenic environment in cancer or may be due to differences in T cell suppression and MDSC isolation protocols. Further studies are needed to determine not only what role mature high-density neutrophils are playing as part of the heterogenous group of cells that are considered PMN-MDSCs, but also their role in steady-state, homeostatic environments.

There are only a few studies that have examined Siglec expression on PMN-MDSCs (30, 31), and these previous studies did not differentiate between the low- and high-density neutrophil fractions. Using our panel, we have shown differential Siglec expression on CD16^low^ versus CD16^high^ low-density neutrophils. It is curious that both the CD16^high^ population and high-density neutrophils had similar Siglec expression patterns in healthy subjects and is what led us to our initial hypothesis that the CD16^low^Siglec9^low^ cells might be the PMN-MDSC population, particularly because of their higher expression of CD33. However, our results clearly revealed that the CD16^low^Siglec9^low^ low-density neutrophils were not suppressive towards CD4^+^ or CD8^+^ T cells, which supports this population as not being PMN-MDSCs. In our suppression assay, we used bead-linked CD3/CD28 antibodies to activate T cells, which is also the method used by most studies. However, some studies use allogenic T cells, as opposed to autologous T cells as we used, which is an important variable that could possibly lead to different outcomes in the T cell suppression assay.

Given the inability of the CD16^low^Siglec9^low^ cells to be immunosuppressive, it brings up two interesting questions: (i) if they are not MDSCs, what are these cells? and (ii) why were their numbers so strikingly increased in cancer patients? Addressing the first question, it is noteworthy that one study isolated HLA-DR^-^CD33^mid^CD14^-^CD15^+^ low-density cells, and then characterized them further using CD10 and CD16 expression, with similar findings as us, in that the CD10^-^CD16^-^ cells were not suppressive (20). Just as interesting, the CD10^+^CD16^+^ cells from this study were just as suppressive as high-density neutrophils, which is also similar to our observations. As CD10 and CD16 are both markers of neutrophil maturation, it is likely that CD10^-^CD16^-^ low-density neutrophils may just be immature neutrophils consisting of myeloblasts, promyelocytes, or myelocytes (7). Another study isolated CD33^mid^CD66b^+^CD14^-^ low-density cells and further characterized them using CD16 (21). This study found that the CD16^+^ population was the most suppressive, while the CD16^-^ cells were least suppressive. Importantly, the CD16^+^ cells had a polymorphonuclear shaped nuclei that is characteristic of a mature neutrophil, while the CD16^-^ cells had a nuclear morphology of a band cell, which is characteristic of an immature neutrophil. These results may indicate that CD16^low^ low-density neutrophils are simply immature neutrophils, while CD16^high^ low-density neutrophils may be mature neutrophils that have degranulated. Addressing the second question, it is well established that PMN-MDSCs are increased in the peripheral blood in a multitude of cancers, which is what made us initially speculate that the CD16^low^Siglec-9^low^ cells might be PMN-MDSCs. We speculate that the increased presence of CD16^low^Siglec-9^low^ low-density neutrophils in the blood of cancer patients is likely due to the neutrophil “left shift” whereby the bone marrow releases more immature neutrophil precursors into the blood in response to infection or other stressors (40–42). It is interesting that only half of the cancer patients had elevated levels of the CD16^low^Siglec-9^low^ population. The wide distribution observed could be due to the stage of disease, as well as the course of treatment that each patient was undergoing. In the future, it would be interesting to follow patients through the course of their disease, to see if CD16^low^Siglec-9^low^ low-density neutrophil fluctuations correlated with increased severity or treatment regime. As we did not have access to fresh blood from cancer patients, the CD16^high^Siglec-9^high^ low-density neutrophil population could not be quantified in cancer patients because the numbers of this population were altered after overnight incubation of the blood at 4°C, which likely represents *ex vivo* activation/degranulation of neutrophils (43, 44).

In summary, we have shown that Siglecs are differentially expressed on CD16^low^ and CD16^high^ low-density neutrophil subsets. T cell suppressive activity tracks with CD16 expression but it is also crucial to point out that regular CD16^high^ high-density neutrophils showed as strong, or even stronger, immunosuppressive capacity. Therefore, density centrifugation of whole blood to separate the low- and high-density cells is an important step that should ideally be carried out when looking for PMN-MDSCs. Our findings underscore the hypothesis that PMN-MDSCs are a difficult-to-pinpoint population of cells, but our definition of Siglec expression on neutrophil subsets should help in this quest.

## Data Availability

The raw data supporting the conclusions of this article will be made available by the authors, without undue reservation.
